# Push Notifications From a Mobile App to Improve the Body Composition of Overweight or Obese Women: Randomized Controlled Trial

**DOI:** 10.2196/13747

**Published:** 2020-02-12

**Authors:** Alberto Hernández-Reyes, Fernando Cámara-Martos, Guillermo Molina Recio, Rafael Molina-Luque, Manuel Romero-Saldaña, Rafael Moreno Rojas

**Affiliations:** 1 Department of Bromatology and Food Technology University of Córdoba Córdoba Spain; 2 Department of Nursing School of Medicine and Nursing University of Córdoba Córdoba Spain; 3 Department of Occupational Safety and Health Córdoba City Hall Córdoba Spain

**Keywords:** exercise, text message, mobile phone, mHealth, health behavior, behavior maintenance, physical activity, push

## Abstract

**Background:**

Technology—in particular, access to the Internet from a mobile device—has forever changed the way we relate to others and how we behave in our daily life settings. In recent years, studies have been carried out to analyze the effectiveness of different actions via mobile phone in the field of health: telephone calls, short message service (SMS), telemedicine, and, more recently, the use of push notifications. We have continued to explore ways to increase user interaction with mobile apps, one of the pending subjects in the area of mHealth. By analyzing the data produced by subjects during a clinical trial, we were able to extract behavior patterns and, according to them, design effective protocols in weight loss programs.

**Objective:**

A clinical trial was proposed to (1) evaluate the efficacy of push notifications in an intervention aimed at improving the body composition of adult women who are overweight or obese, through a dietary procedure, and (2) analyze the evolution of body composition based on push notifications and prescribed physical activity (PA).

**Methods:**

A two-arm randomized controlled trial was carried out. A sample size of 117 adult obese women attended a face-to-face, 30-minute consultation once a week for 6 months. All patients were supplied with an app designed for this study and a pedometer. The control group did not have access to functionalities related to the self-monitoring of weight at home, gamification, or prescription of PA. The intervention group members were assigned objectives to achieve a degree of compliance with diet and PA through exclusive access to specific functionalities of the app and push notifications. The same diet was prescribed for all patients. Three possible PA scenarios were studied for both the control and intervention groups: light physical activity (LPA), moderate physical activity (MPA), and intense physical activity (IPA). For the analysis of three or more means, the analysis of variance (ANOVA) of repeated means was performed to evaluate the effects of the intervention at baseline and at 3 and 6 months.

**Results:**

Receiving notifications during the intervention increased body fat loss (mean -12.9% [SD 6.7] in the intervention group vs mean -7.0% [SD 5.7] in the control group; *P*<.001) and helped to maintain muscle mass (mean -0.8% [SD 4.5] in the intervention group vs mean -3.2% [SD 2.8] in the control group; *P*<.018). These variations between groups led to a nonsignificant difference in weight loss (mean -7.9 kg [SD 3.9] in the intervention group vs mean -7.1 kg [SD 3.4] in the control group; *P*>.05).

**Conclusions:**

Push notifications have proven effective in the proposed weight loss program, leading women who received them to achieve greater loss of fat mass and a maintenance or increase of muscle mass, specifically among those who followed a program of IPA. Future interventions should include a longer evaluation period; the impact of different message contents, as well as message delivery times and frequency, should also be researched.

**Trial Registration:**

ClinicalTrials.gov NCT03911583; https://www.clinicaltrials.gov/ct2/show/NCT03911583

## Introduction

Mobile technology can be considered as being fundamental to our lives. Its presence is ubiquitous; estimates in 2019 have suggested that there will be around 4.1 billion intelligent devices globally, between mobile phones and tablets [1]. Furthermore, the wearable market is also a promising one, since the number of wearable devices connected worldwide is expected to jump to over 1.1 billion in 2022, while telecom technology will change from 4G to 5G [2]. Furthermore, this fact has changed the way we relate to each other, live, and work [3]. In 2014 there was a turning point in connectivity; for the first time, the amount of browsing through mobile devices exceeded that of desktop computers [4]. We can, therefore, dispense with the adjective *mobile* when we talk about *technology*, as both terms are already inseparable [3].

The health care field has not been alien to this revolution. The mHealth concept was born in 2000; the 2010 mHealth Summit held by the Foundation for the National Institutes of Health defined it as “the provision of health care services through mobile communication devices” [5]. Nowadays, around 40% of the 300,000 apps available in the major app stores are related to health, particularly those focused on disease monitoring and management [6].

Communication technology has evolved, taking us from making phone calls or sending short message service (SMS) text messages to developing telemedicine via Web or mobile apps as supports for clinical decision making or increasing the degree of adherence to treatments [7-9]. SMS text messaging has shown itself to be a great resource for delivering electronic reminders in practice and a highly feasible platform, since it is an older technology that can be used on any existing mobile phone. It has shown clear benefits in increasing adherence to treatment [10], preventing complications in chronic conditions [11], allowing communication between professionals [12], and helping in disease self-management [13], among others. In these situations, SMS text messaging has been used alone [11,12] or in combination with other technologies [13,14].

Push technology, however, has recently emerged in the mHealth sector because of its potential for improving pervasive functionalities in mobile health apps. It permits the delivery of timely updates and customized reminders to its users, with respect to the time sent and their contents. A push notification has been defined as being an event-based mechanism by which remote servers *push* events to mobile phone client apps [15]. This functionality offers auditory and visual alerts to inform users about an incoming message and invites them to act, even if the app sending the notifications is not currently in use [16]. Push notifications have proven to be effective in communication with professionals [17] and assessing health behavior patterns [18], but there is scant evidence of their effectiveness in interventions aimed at changing lifestyles.

Recent reviews confirm that providing digital solutions to people with an interest in health interventions improves the results obtained [19,20]. Some functions, such as self-monitoring, interaction between users, or setting objectives, have positive effects on health status [21]. In particular, self-evaluation is a characteristic that has shown encouraging results and has been the subject of study in clinical trials [22,23].

The role of physical activity (PA) in weight loss programs, as well as in the maintenance of weight loss in the long term, is fundamental [24] and has been confirmed in recent years through several systematic reviews [25,26]; this has revealed the existence of an inverse association between PA and body mass index (BMI).

Taking all of the above into account, a clinical trial was proposed to evaluate, as a primary outcome, the efficacy of push notifications in an intervention aiming to improve body composition—defined as loss of fat mass while maintaining or increasing muscle mass—of adult women who are overweight or obese. Assessing the evolution of body composition based on diet and prescribed PA was considered a secondary outcome.

## Methods

### Study Design: Overview

A two-arm randomized controlled trial of a 6-month intervention (ie, prescription of PA and diet) was carried out. The intervention group was comprised of women who received push notifications, while women assigned to the control group did not receive any. All the women in both groups followed the same diet. In addition, the women in the intervention group and the control group were randomly assigned to programs of PA of different intensities: light physical activity (LPA), moderate physical activity (MPA), or intense physical activity (IPA). The intervention group received push notifications with the aim of establishing a mechanism of control, gamification, and reminders of the PA prescribed in a face-to-face consultation. The control group, despite being recommended and prescribed corresponding PA, did not have a control and monitoring mechanism associated with push notifications. After enrollment, body composition variables were assessed every week for 24 weeks. The study protocol complied with the Declaration of Helsinki for medical studies and was approved by the bioethical committee of the University of Córdoba from the Department of Health at the Regional Government of Andalusia (Act No. 284, reference 4156). The protocol was registered at ClinicalTrials.gov (NCT03911583). This trial has been reported according to the Consolidated Standards of Reporting Trials (CONSORT) statement and the CONSORT-Electronic and Mobile HEalth Applications and onLine TeleHealth (EHEALTH) extension (see Multimedia Appendix 1).

### Recruitment and Enrollment

The sample consisted of 117 Caucasian women from the region of Andalusia, Spain. Participants were recruited from a private health center, to which they came on their own initiative to undergo a weight loss program; women learned of the program either through ads published within the clinic itself or specific publicity in social networks. Data began to be collected on January 1, 2016, and lasted for a period of 2 years. All study participants were required to sign a written informed consent form.

### Randomization

Participants were randomly assigned following a simple randomization procedure (ie, computerized random numbers) to intervention or control groups and to LPA, MPA, or IPA groups. Participants were randomized using a random-number generator in Microsoft Excel (Microsoft Corporation‬).

### Sample Size Calculation

The primary outcome variable was fat mass loss after 6 months; the anticipated minimum difference in the average fat mass loss was 2% with an expected SD not exceeding 3.5% [27]. The study was designed to have at least 80% power and an alpha level set at .05, obtaining a sample size of 27 individuals in each of the intervention and control groups for a total of 54. A total of 90 women—45 in each group—was estimated to be necessary to mitigate the effect of possible dropouts during this trial.

### Push Notifications

**Figure 1:**
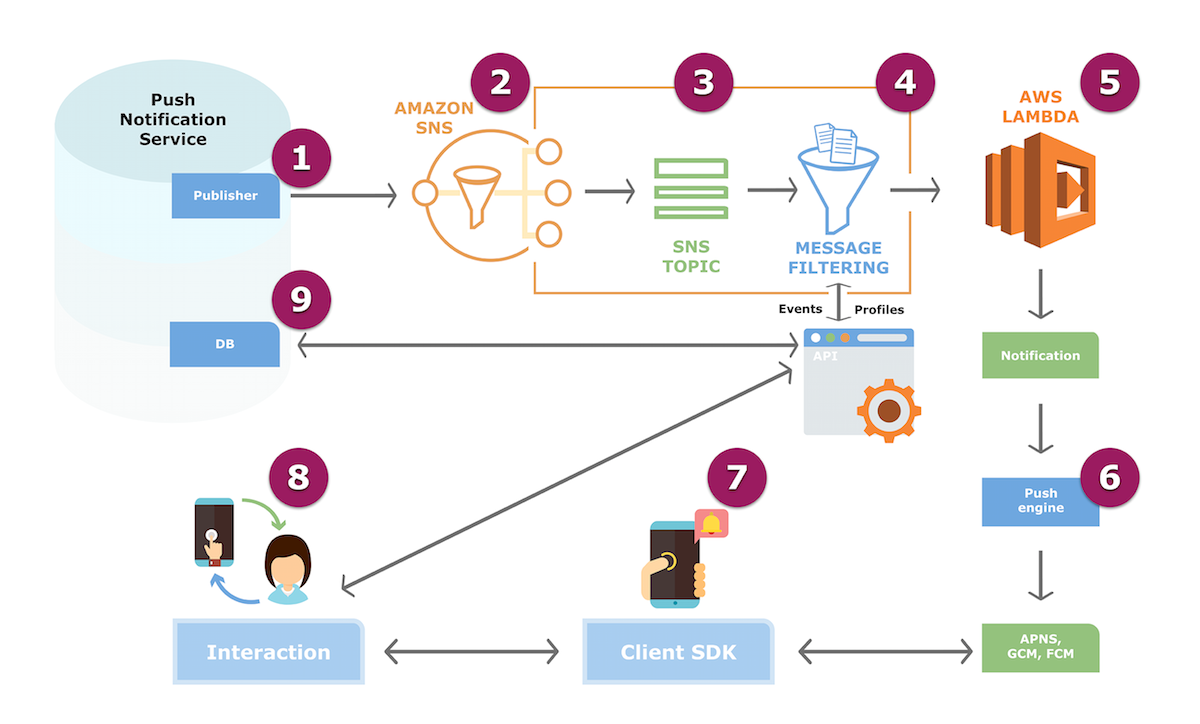
Push notification flow. The push notification service system works under the Amazon Simple Notification Service (SNS), which provides topics for push-based and many-to-many messaging. Using Amazon Web Services (AWS) Lambda functions from the SNS, messages are sent to a large number of subscriber end points via parallel processing. This process consists of eight steps. Step 1. The publisher sends push notifications from distributed systems. Step 2. Amazon SNS is activated to get fully managed publisher and subscriber messaging and event-driven computing service, including steps 3 and 4. Step 3. SNS Topic: message publishers are decoupled from subscribers by topic. Step 4. Message Filtering: messages are filtered according to subscription filters, which allows for personalization, and are delivered to clients, who connect to the database (DB) through an application programming interface (API). Step 5. AWS Lambda creates notifications and sends them to the client software development kit (SDK) engine through the Apple Push Notification service (APNs), Google Cloud Messaging (GCM), and Firebase Cloud Messaging (FCM). Step 6. The client SDK receives push notifications. Step 7. The user interacts with the push notification. Step 8. The interaction is recorded in the DB through the app’s API.

Automatic push notifications (see Figure 1) were scheduled to be sent to the intervention group on specific days with personalized health-related and motivational messages; these messages aimed to provide comments to reinforce behavior modification and encourage interaction with the app.

The content of the feedback messages was extracted from a previously established library (see Textbox 1 and Multimedia Appendix 2) and was based on the following behavioral theories:

1. Health tips, where the primary tailoring goals are attention and peripheral processing [28].
2. PA tips, in this case attention and being informed [29].
3. Self-monitoring tips, where the primary tailoring goals are decision making and behavioral intention [30].

**Main themes of the app message library.**

- Health tips
- Nutritional properties of specific foods
- Healthy options when it comes to selecting snacks
- Physical activity tips
- Examples of healthy habits
- Benefits of carrying out some protocols periodically
- Self-assessment tips: includes a specific menu for users to enter their weight at home—exclusive option for the intervention group

### Randomized Test Design

**Figure 2:**
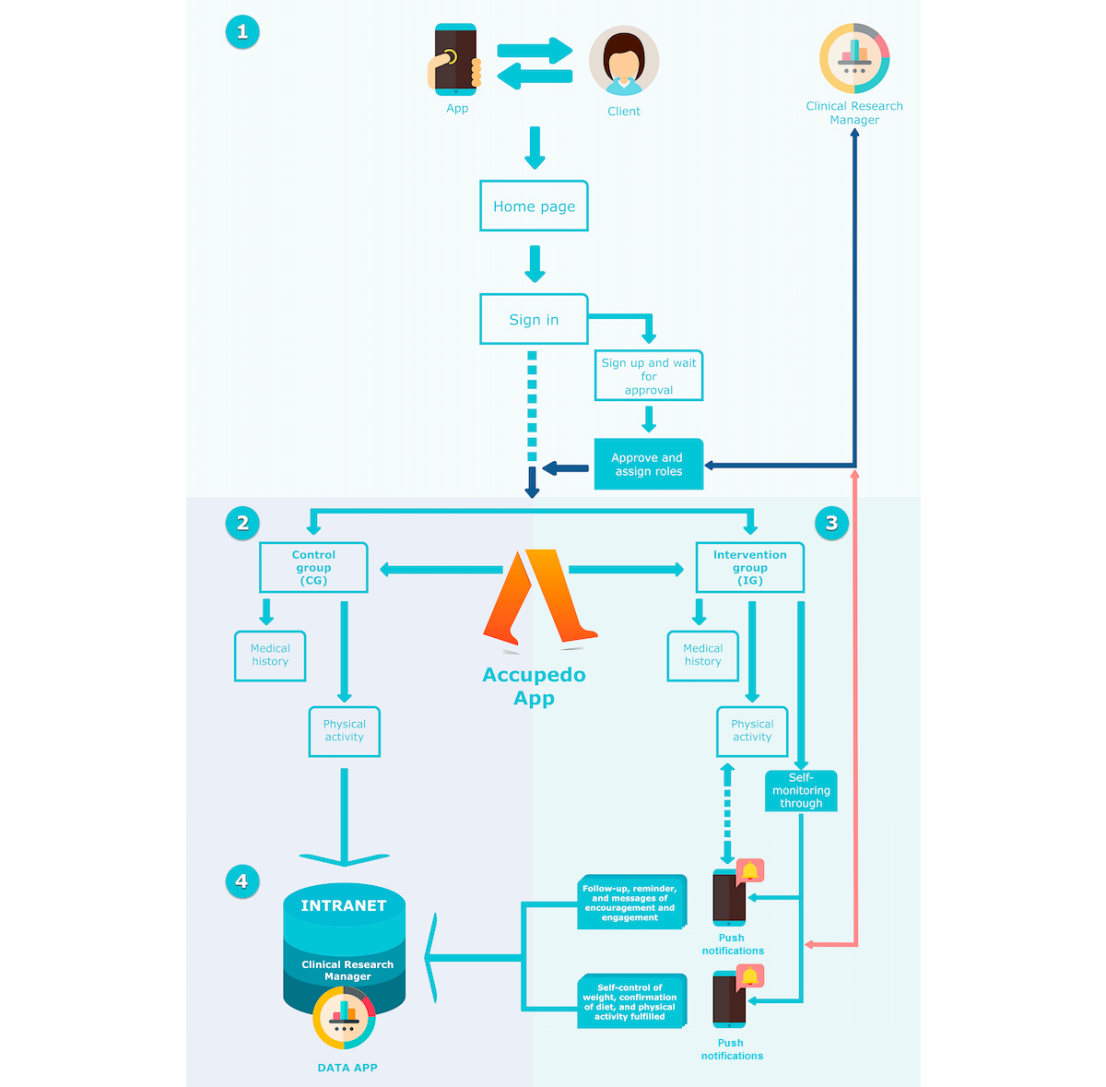
Implementation of push notifications in the study design. Step 1. Clients log in and the system recognizes the group to which each woman was assigned (control or intervention group). Step 2. Women in the control group are given access to their electronic medical record (ie, evolution in anthropometric indicators) and can register their physical activity (PA), including daily steps measured with the Accupedo app. Step 3. Women in the intervention group are given access to the same functionalities as those in the control group. In addition, they receive push notifications to increase self-control (ie, reminders, support messages, and request for registration of compliance with the dietary and PA plans). Step 4. All data are received and recorded in the Clinical Research Manager (Intranet).

The implementation of the methodology for sending push notifications (see Figure 2) was designed considering aspects collected in previous studies that mention, above all, the importance of the following: (1) patients’ ability to select their preferred time for receiving notifications and (2) the increased effectiveness of notifications when delivered at times that do not interrupt the daily routine [31]. Based on these criteria, three time points were established at which the notifications would be sent to the patients in the intervention group: 08:30 (point 1)—the time point before going to work; 14:00 (point 2)—lunch time; and 20:00 (point 3)—the time when patients arrived home, before dinner. The first message was sent between points 1 and 2, according to the patients’ preferences; patients who did not respond to the push notifications automatically received a notification at point 3.

The notifications were sent from an online tool developed specifically for this study. This tool allows the researcher to program push notifications and to check for who has responded and when (see Multimedia Appendix 3).

### Outcome Measures

#### Physical Activity

To estimate the degree of PA and sedentary activity at the beginning of the study, we used the long version of the International Physical Activity Questionnaire (IPAQ-long), which has been shown to be reliable and valid for estimating PA and sitting-down time [32]. To adjust for sedentary behavior and/or PAs outside working hours, the IPAQ-long was administered via interview at the beginning of the study and repeated at the end of the intervention. The Accupedo app—a pedometer app—was installed on the patients' mobile phones. This app is capable of tracking and storing information about daily PA in relation to walking or running; as well, it has been previously validated as a measure to encourage and motivate patients to reach a certain number of steps [33]. The purpose of the push notifications in this case was to encourage and remind patients of the objective that was previously established in the face-to-face consultation, regarding the number of steps. Patients had to report, within the app, the data obtained in Accupedo; this information was checked weekly in the consultation by the research team.

#### Self-Reporting

**Figure 3:**
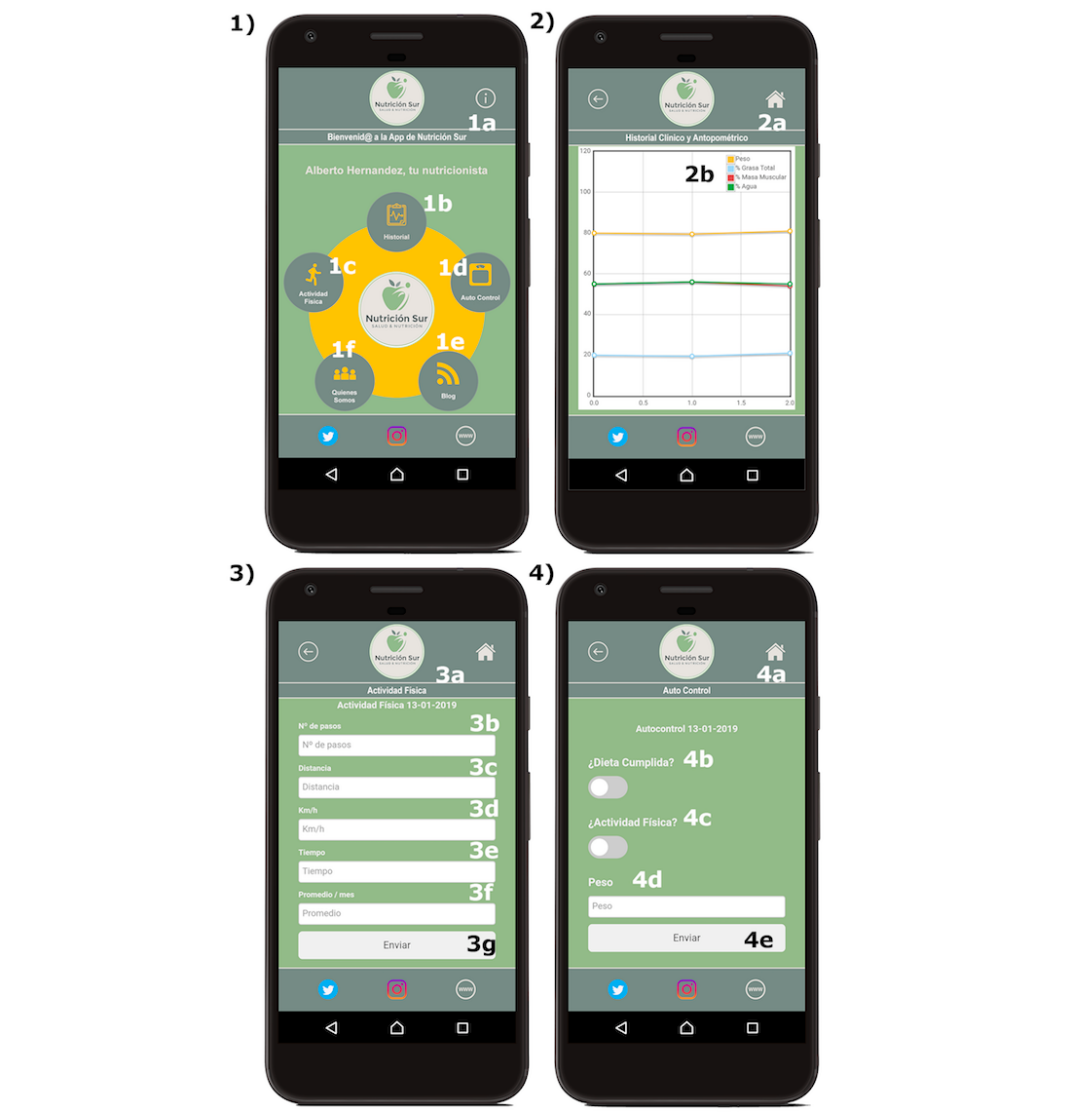
Screenshots of the full version of the app, including the self-control functionality for the intervention group. 1. Screenshot of the main menu. The following are translated from Spanish: 1a. Welcome to the Nutrición Sur app; 1b. Medical history; 1c. Physical activity; 1d. Self-control; 1e. About us; 1f. Blog. 2. Screenshot of the electronic medical record. The following are translated from Spanish: 2a. Medical and anthropometric history; 2b. Weight (orange), Total fat (blue), Muscle mass (red), Total water (green). 3. Screenshot of the physical activity record. The following are translated from Spanish: 3a. Physical activity; 3b. Number of steps; 3c. Distance; 3d. km/h; 3e. Time; 3f. Average/month; 3g. Submit. 4. Screenshot of the self-control page. The following are translated from Spanish: 4a. Self-monitoring; 4b. Diet fulfilled?; 4c. Physical activity?; 4d. Weight; 4e. Submit.

Self-evaluation was related to the women’s behavior during each week. The women had to enter their weight in the app when they received the push notification. The objective was to determine whether receiving a reminder and keeping track of weight made a difference among the women in the intervention group compared to the group of patients without access to this functionality (see Figure 3). Apps that offer self-evaluation features have previously demonstrated to help patients lose weight [34].

### Eligibility Criteria: Inclusion and Exclusion

**Figure 4:**
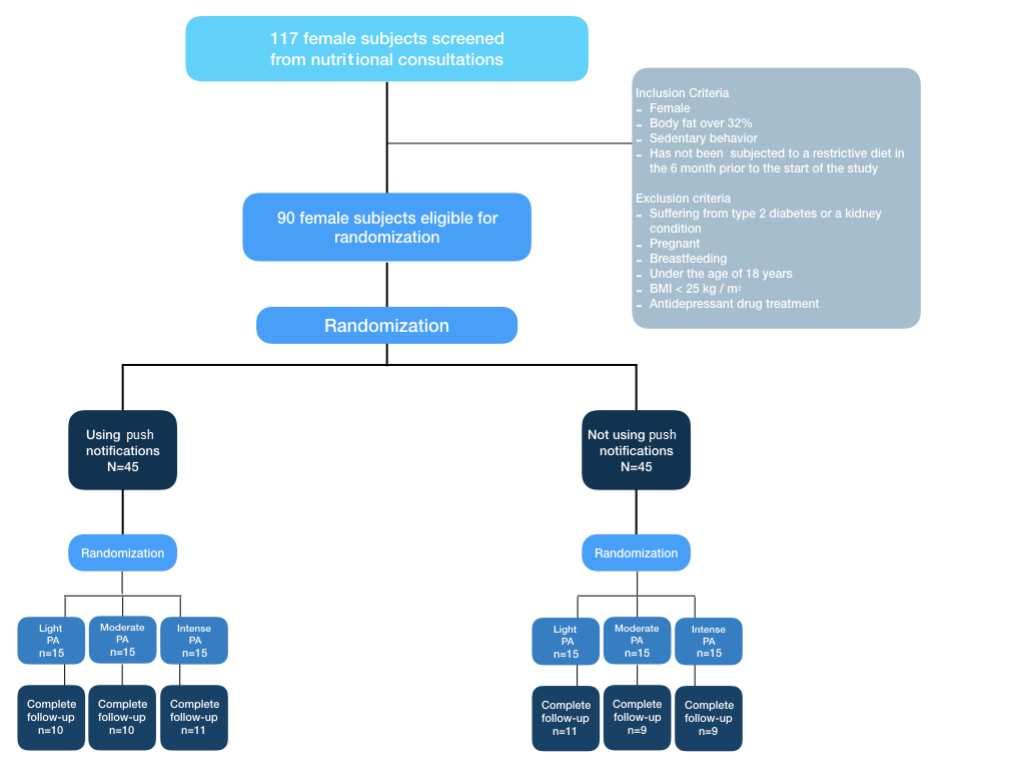
Flowchart of participants. BMI: body mass index; PA: physical activity.

A flowchart of participants is shown in Figure 4. Of the 117 women who attended the consultations to lose weight, 27 (23.1%) did not meet the inclusion criteria, so the remaining 90 women (76.9%) were randomized into two groups: intervention group or control group. After this, the women in each group were randomly assigned to one of the three PA programs described previously. We decided to study a group of women, exclusively, because of differences from men with respect to body composition (ie, a higher percentage of fat mass) and metabolic response to PA plans (ie, lower capacity to build muscle mass) [35]. For this reason, and since the primary objective was to analyze the effect of push notifications, we decided not to include men. Another inclusion criterion was having at least 32% of total body fat at the start of the study because this is the lowest limit that determines obesity in a woman, as the percentage of fat is considered a marker of obesity [36].

Women who had a metabolic illness and those who had previously been diagnosed with type 2 diabetes mellitus were excluded from the study. Exclusion criteria also included being pregnant or breastfeeding, being under the age of 18, and having a BMI of less than 25 kg/m^2^. Finally, another exclusion criterion was taking antidepressant drugs, due to their possible role in the incidence of obesity in middle-aged women [37]. A total of 60 women out of 90 completed the follow-up evaluation, representing a retention rate of 67%. There were no statistical differences in the dropout rate between any of the groups.

### Study Variables and Measurements

Body fat, muscle mass, and water percentages, considering variable outcomes, were monitored and collected over time using multifrequency bioelectrical impedance with the BWB-800A electronic scale (Tanita Corporation of America), which was previously validated [38]. Standing height without shoes was measured to the nearest millimeter using the wall-mounted PORTROD stadiometer (Health o meter). The independent variables collected were age (years), height (cm), weight (kg), and BMI (kg/m^2^). The anthropometric measurements were collected following the recommendations of the reference manual of standardized anthropometry [39].

For the PA, the strata proposed by Matthew were used [40]. Patients in MPA and IPA groups were instructed to perform aerobic exercises corresponding to energy expenditure induced by training of approximately 300-600 kcal/day. For PA to be considered MPA, women had to walk for 30-60 minutes every day or complete 7500-10,000 steps per day. On the other hand, to be considered IPA, patients had to carry out, in addition to the target set in the MPA group, IPA sessions of over 70% of VO_2_max three times a week. Several members of the research team—nurses and a nutritionist—assessed the women in order to prescribe the adequate intensity of PA. This was based on heart rate, estimated using the Karvonen formula [41], and establishing the maximum heart rate of 220 - age (years). Adherence was monitored by weekly exercise records that were completed by participants and researchers.

With respect to diet, the daily energy requirements were determined by estimating the energy expenditure at rest through the formula proposed by Harris-Benedict (655.0955 + 9.5634 [weight in kg] + 1.8496 [size in cm] - 4.6756 [age in years]) [42] and multiplying the value obtained by a factor of 1.5 in those patients who did PA [43]. During a period of 24 weeks, all the participants followed a diet with the following distribution of macronutrients: 25%-30% protein, 40%-45% carbohydrates, and 30%-35% fat. A hypocaloric diet was designed, with a reduction of 500 kcal/day during the intervention period to achieve a weekly weight loss of 400 grams; no vitamins or other nutritional supplements were prescribed. In order to be included in the study, each woman participated in a 1-hour seminar, in which a dietitian-nutritionist taught her the appropriate selection and preparation of food. The proposed menu was valid for 7 days, and at the weekly review appointment, the protocol for the current week was handed out. The energy and nutritional contributions were assessed through the Dietowin program and the weighing method [44].

The follow-up tests began during the first week of the diet and PA assignment. Body composition was measured after an overnight fast; the subject was then required to go to the center on the same day of the week at the same time, wearing the same clothes. Review appointments continued on a weekly basis until week 24, when all the variables were collected.

### Statistical Analysis

The quantitative variables have been presented with the mean and SD, and the qualitative variables with frequencies and percentages.

To contrast the goodness of fit with a normal distribution of data from quantitative variables, the Kolmogorov-Smirnov test with the Lilliefors correction was used. For the bivariate hypothesis, the Student's *t* test was performed for two means, while for the qualitative variables the chi-square and Fisher exact tests were employed when necessary. Likewise, for the analysis of three or more means, the analysis of variance (ANOVA) of repeated means determined the effects of the intervention at baseline and at 3 and 6 months; the correlation between the quantitative variables was verified by the coefficient of Pearson correlation (r). Finally, if the normality or homoscedasticity criterion was not met for the ANOVA, the Kruskal-Wallis test was performed.

To adjust for the possible impact of PA on body composition and its possible role as a confounding factor, adjusted linear regressions were made for each body composition variable (percent body fat and muscle mass) and weight, calculating the standardized beta coefficients. To determine the goodness of fit of the models, the SE, the adjusted coefficient of determination, the F statistic, the linearity, and the residuals were analyzed.

For all statistical analyses, an alpha error of less than 5% was accepted (*P*<.05) and a 95% CI was calculated. For the statistical analysis, IBM SPSS Statistics software, version 22.0 (IBM Corp), was used.

## Results

### Characteristics of the Population Studied

The women studied had a mean age of 41.5 years (SD 11.3). With regard to body composition, in the first consultation we found an average weight of 82.6 kg (SD 14.5) (95% CI 78.8-86.3), average muscle mass of 44.7 kg (SD 5.1) (95% CI 43.4-46), and average body fat of 42.2% (SD 5.5) (95% CI 40.8-43.6) (see Table 1).

**Table 1:** Descriptive characteristics of participants randomized at baseline.

Variable	Total (N=60), mean (SD)	No push notifications (n=29), mean (SD)	Push notifications (n=31), mean (SD)	*P* value
Age (years)	41.5 (11.3)	40.3 (11.6)	42.9 (10.9)	.38
Height (m)	1.6 (0.1)	1.6 (0.1)	1.6 (0.1)	.77
Weight (kg)	82.6 (14.5)	84.8 (14.9)	80.5 (13.9)	.25
Body mass index (kg/m^2^)	31.8 (5.3)	32.8 (5.3)	31.0 (5.3)	.19
Body fat (%)	42.2 (5.5)	43.4 (5.0)	41.0 (5.8)	.10
Muscle mass (kg)	44.7 (5.1)	45.0 (5.3)	44.4 (4.9)	.66
Water (%)	43.1 (3.9)	42.1 (3.3)	44.1 (4.2)	.05

No significant differences were found between the group that received notifications and the one that did not, with respect to the number of women who remained in the LPA group or engaged in MPA or IPA (*P*>.05). The baseline data according to whether the push notifications were sent or not are shown in Table 1.

### Analysis of the Evolution of Body Composition Based on the Use of Push Notifications

The analysis of the variation in body composition in the study groups was based on the percentage changes between the measurements collected at baseline and at 3 and 6 months. A total of 3 months after the intervention, the group that received push notifications showed a significantly greater reduction in the percentage of body fat (-8.4% [SD 4.7], 95% CI -10.1 to -6.6; *P*=.005). For the rest of the anthropometric variables, although there was a greater decrease in the intervention group, the decreases were not significant with respect to the control group (*P*>.05).

However, this trend was not maintained after 6 months of the intervention. The women who received push notifications showed a greater improvement in their body composition than those who did not receive them; a higher decrease in body fat percentage (*P*<.001), a smaller reduction in muscle mass (*P*<.05), and a higher percentage increase in body water (*P*<.05) were observed (see Table 2).

**Table 2:** Variation of body composition.

Variable	At 3 months			At 6 months		
	No push notifications, mean (SD)	Push notifications, mean (SD)	*P* value	No push notifications, mean (SD)	Push notifications, mean (SD)	*P* value
Weight (kg)	-6.3 (3.3)	-7.1 (2.4)	.27	-7.1 (3.4)	-7.9 (3.9)	.39
Body mass index, (kg/m^2^)	-2.1 (1.2)	-2.3 (1.0)	.56	-8.0 (3.7)	-9.1 (5.7)	.36
Body fat (%)	-5.0 (4.2)	-8.4 (4.7)	.005	-7.0 (5.7)	-12.9 (6.7)	<.001
Muscle mass (kg)	-2.6 (3.1)	-1.6 (4.1)	.27	-3.2 (2.8)	-0.8 (4.5)	.02
Water (%)	3.2 (3.3)	5.1 (4.5)	.07	4.8 (4.3)	8.0 (5.8)	.02

### Analysis of the Evolution of Body Composition Based on the Use of Push Notifications and Physical Activity

At 3 months, it was found that there was no significant change in weight, nor in BMI or muscle mass, in women who remained in the LPA group. However, we observed a reduction in body fat percentage in women who received motivational messages during the intervention (-5.9% [SD 2.3], 95% CI -7.5 to -4.2) and a significantly elevated water percentage (3.4% [SD 1.3], 95% CI 2.4-4.3), compared to that in women who did not receive the messages.

With regard to women who performed some type of physical activity (ie, MPA and IPA), although a more favorable change was found among the women who received the push notifications, it was not significant (*P*>.05) (see Table 3).

The trend varied in the data collected at 6 months among the three study groups. Regarding sedentary women, it was found that the difference in body fat loss and water gain was significantly greater (*P*<.001 and *P*<.01, respectively) among women who received push notifications.

With respect to the group that did MPA, women in the control group showed greater modifications in all body composition variables, but they were only significant in the case of body fat (*P*<.01) (see Table 4).

**Table 3:** Evolution of body composition based on physical activity (PA) and push notifications at 3 months.

Variable	Light PA (n=21)				Moderate PA (n=19)				Intense PA (n=20)		
	No push notifications (n=11), mean (SD)	Push notifications (n=10), mean (SD)	*P* value	No push notifications (n=9), mean (SD)		Push notifications (n=10), mean (SD)	*P* value	No push notifications (n=9), mean (SD)		Push notifications (n=11), mean (SD)	*P* value
Weight (kg)	-4.8 (3.8)	-6.5 (2.0)	.22	-6.9 (2.9)		-8.1 (1.7)	.36	-7.5 (2.6)		-6.7 (3.2)	.66
Body mass index (kg/m^2^)	-1.7 (1.5)	-2.1 (0.7)	.56	-2.3 (0.8)		-2.5 (0.9)	.84	-2.3 (0.9)		-2.2 (1.3)	.55
Body fat (%)	-2.3 (3.6)	-5.9 (2.3)	.02	-5.6 (3.5)		-6.8 (2.4)	.60	-8.0 (3.5)		-12.0 (5.7)	.07
Muscle mass (kg)	-2.8 (3.9)	-2.9 (2.4)	.71	-2.9 (2.3)		-3.7 (1.8)	.55	-2.1 (2.8)		1.6 (5.0)	.11
Water (%)	1.5 (2.5)	3.4 (1.3)	.04	3.1 (3.7)		4.2 (1.4)	.07	5.6 (2.4)		7.6 (6.9)	.30

**Table 4:** Evolution of body composition based on physical activity (PA) and push notifications at 6 months.

Variable	Light PA (n=21)			Moderate PA (n=19)			Intense PA (n=20)			
	No push notifications (n=11), mean (SD)	Push notifications (n=10), mean (SD)	*P* value	No push notifications (n=9), mean (SD)	Push notifications (n=10), mean (SD)	*P* value		No push notifications (n=9), mean (SD)	Push notifications (n=11), mean (SD)	*P* value
Weight (kg)	-5.6 (3.1)	-7.2 (1.9)	.07	-9.5 (2.3)	-11.4 (2.4)	.13		-10.0 (3.7)	-10.0 (4.3)	>.99
Body mass index (kg/m^2^)	-5.3 (2.6)	-7.1 (2.0)	.09	-9.6 (2.3)	-9.5 (5.2)	.54		- 9.5 (4.3)	-10.6 (7.9)	.88
Body fat (%)	-1.2 (1.5)	-6.2 (2.1)	<.001	-8.1 (2.6)	-12.8 (2.6)	.002		-13.0 (3.7)	-19.0 (6.1)	.046
Muscle mass (kg)	-4.3 (2.2)	-3.0 (2.1)	.28	-3.6 (2.1)	-2.9 (1.9)	.24		-1.4 (3.4)	3.0 (5.3)	.08
Water (%)	1.2 (1.8)	4.1 (1.6)	.003	4.6 (3.2)	8.2 (2.1)	.02		9.3 (3.1)	11.4 (8.1)	.60

Finally, women who were referred to IPA and received push notifications had significantly greater reductions in the percentage of body fat (received notifications: -19.0% [SD 6.1], 95% CI -23.1 to -15; did not receive notifications: -13.0% [SD 3.7], 95% CI -15.5 to -10.5; *P*<.05). On the other hand, although no significant differences in muscle mass were found, muscle mass gain was observed in the intervention group.

### Adjusted Regressions in Body Composition Modifications

The results shown in Table 5 confirm that the incorporation of push notifications had a different impact in body composition variables. Thus, we observed how receiving these notifications during the intervention lead to the weight loss increase (standardized beta=-.208) and helped to maintain or gain muscle mass (standardized beta=.266). However, the most important impact was observed on body fat, where loss occurred to a high degree (standardized beta=-.397).

**Table 5:** Multiple linear regression models.

Result variable and models^a^ and measures they are adjusted for			Standardized beta	*R* ^2b^	SE	*r* ^c^	*P* value
**Weight lost at 6 months (kg)**							
	**2.214 - 4.335 (MPA^d^- 4.219 (IPA^e^) - 1.487 (push notifications^f^) - 0.092 (weight at baseline)**			**.393**	**2.812**	**.658**	**<.001**
		MPA	-.564				
		IPA	-.556				
		Push notifications	-.208				
		Weight at baseline	-.367				
**Body fat lost at 6 months (%)**							
	**1.443 - 6.720 (MPA) - 12.390 (IPA) - 5.379 (push notifications) - 0.058 (body fat at baseline)**			**.743**	**3.461**	**.872**	**<.001**
		MPA	-.462				
		IPA	-.863				
		Push notifications	-.397				
		Body fat at baseline	-.047				
**Muscle mass lost at 6 months (kg)**							
	**5.769 - 0.029 (MPA) + 3.926 (IPA) + 2.062 (push notifications) - 0.226 (muscle mass at baseline)**			**.416**	**2.985**	**.675**	**<.001**
		MPA	-.003				
		IPA	.478				
		Push notifications	.266				
		Muscle mass at baseline	-.294				

^a^Models are adjusted for age, weight at baseline, push notifications, percentage of fat at baseline, muscle mass at baseline, percentage of water at baseline, and physical activity (PA).

^b^*R*^2^: coefficient of determination (goodness of fit).

^c^*r*: Pearson´s linear correlation.

^d^MPA: moderate physical activity (sedentary=0, moderate=1).

^e^IPA: intense physical activity (light=0, intense=1).

^f^Push notifications (no=0, yes=1).

## Discussion

### Principal Findings

As far as we know, this work has been the first clinical trial to study the impact of establishing a tracking and gamification system through push notifications in a weight loss program that included PA, combined with dietary treatment in face-to-face consultations, with a weekly tracking frequency over 6 months. From the results, we have extracted encouraging data. Introducing an mHealth strategy in patient consultation was shown to be, at the end of the trial period, a differentiating element: women receiving push notifications lost more weight, lost more total body fat, and had better muscle mass results than those who did not receive these notifications.

### Comparison With Prior Work

#### Effects After 12 Weeks of the Push Notifications

In the first 3 months of the study, we found that push notifications only had an effect on total body fat and not on the rest of the anthropometric variables.

In the LPA groups, which only encouraged complying with the diet and carrying out weight control at home, push notifications triggered greater body fat loss, compared to the group who did not receive notifications. What differentiated the patients of this group was their access via the app to a specific functionality called *self-monitoring*, a concept that allows the patient to restrict or cancel a response, which makes decision making possible [45]. The use of technology in a clinical trial of behavioral weight loss in a face-to-face intervention has previously been documented in the work of Polzien et al [46], who reported additional weight loss at 12 weeks of 2.1 kg in the group that had technological support; these data approximate those obtained in our study, since women who received push notifications lost 1.76 kg of additional weight in the LPA group.

We found important differences in applying mHealth technology in combination with face-to-face follow-ups when compared to resorting exclusively to an mHealth intervention. At 12 weeks, our trial subjects lost an average of 7.1 kg (SD 2.4), while participants in another intervention using only text messages lost an average of 1.6 kg (SD 2.6) [47].

Sending messages of encouragement results in an increase in PA in the short term. Although the data in the first 12 weeks show an improvement in the body composition of women who received notifications, this was not significant (*P*>.05). A previous study among sedentary women confirmed that an mHealth intervention helped increase PA, although there were no significant changes in BMI [48].

#### Effects After 24 Weeks of the Push Notifications

At 6 months, the women in the trial who received push notifications lost more fatty tissue and their fat-free mass behaved better (ie, muscle mass and total body water).

A study focusing on the effects of mobile phone-based support and weight loss found a difference in additional weight loss of 1.9 kg at 6 months between groups with and without technology [49]. This difference was greater than the 1.63 kg between women who did or did not receive push notifications in our study, despite the fact that patients were aware that they were being monitored. In previous studies, this aspect was seen to affect patients’ behavior [50]. Our findings were similar to those obtained in the review by Hutchesson et al [51], who compared weight loss among participants assigned to an mHealth intervention (13 studies), finding that the additional characteristics led to an average weight loss difference of 1.46 kg.

The body composition variables of groups assigned to performing MPA and IPA improved, highlighting an increased loss of total body fat and better behavior in fat-free mass (ie, maintaining or gaining). Text messages (ie, push notifications) aimed at fulfilling an assigned PA protocol were effective in interventions of at least 6 months in duration. These results do not coincide with a study of the same duration [52], in which there was no monitoring in face-to-face consultations. Reinforcing the strategy to be followed in the consultation and using technology as a support may be more feasible. However, we found similar results in a study where subjects were monitored at a weekly frequency for 24 weeks [53].

The same conclusion made at 12 weeks on the effectiveness of text messaging and weight loss without face-to-face control can be extrapolated to longer periods of 24 weeks. Even though, in our study, patients monitored through push notifications and face-to-face consultations lost an average of 7.87 kg (SD 3.87), in studies that exclusively measured the effectiveness of mobile messaging and weight loss, the results were not so promising, as participants only lost an average of 1.27 kg (SD 6.51) [54,55].

#### Body Composition

Previous studies reported that decreases in muscle mass were restored over time after weight loss interventions [56,57]. Although the muscle mass loss was lower than that of total body fat, muscle maintenance should be monitored and prescribed even in weight loss programs. The subjects of our study who had a light or moderate exercise prescription (ie, walking) lost muscle mass at the end of the period. The explanation is that this kind of PA seems insufficient for mobilizing and stimulating muscle mass [58]. The group in our study that had an intense physical exercise prescription (ie, incorporating resistance training) was the only one that showed muscle mass gain at 6 months. These results provide evidence that a combined program of aerobic and resistance-type exercise helps to preserve muscle mass during weight loss, results that matched those of a recent review [59].

Our findings reveal that the prescription of PA results in significant body fat loss; the higher the intensity of PA, the greater the loss of fat at 6 months. While the LPA prescription implied a 6% fat loss at 6 months, the IPA subjects reached a fat loss of 19%. In addition, when analyzing the results among MPA and IPA subjects, we observed that IPA subjects lost an additional 7% of fat, whereas no significant weight loss was observed between the MPA and IPA groups. Our results are consistent with the existing literature [60]; although high-intensity training did not improve weight loss over 6 months compared to a lower intensity, the impact on fat loss was significant [61].

### Limitations and Strengths

Although the sample size in this study is similar to that used in previous works [62,63], we carried out a randomization procedure that led to balanced arms; besides, the dropout pattern at 3 months was similar in the three groups. To avoid self-report bias, which was previously documented [64], the data collection records were checked by the research staff in face-to-face consultations on a weekly basis, in which women had to show her Accupedo status directly from their mobile phones. Key strengths include the use of an objective measure of PA, which has stronger associations with health behaviors than hypothetical methods and self-reported measures [65]. To understand the results in body composition and to be able to apply them in public health, our study included a combination of diet and PA and we had to demonstrate the difference in the change in muscle mass between diet, PA programs, or both [58]. In this work, we aimed to contribute to the growing interest in the field of mHealth, regarding improvement of body composition and the change in dynamics of disease prevention and treatment, through text messaging interventions [66]. This study abandons SMS technology to enter into a scenario of greater interaction between patients and their health, allowing feedback from the patients.

Although the number of women who completed the follow-up evaluation is greater than the estimated minimum sample size, the results should be interpreted with caution. In this sense, subsequent investigations with longer follow-up times, larger sample sizes, and with similar designs that allow us to corroborate the effectiveness found in this research would be necessary. Besides, more empirical research is needed to examine the effect of notification content and delivery times, as well as the purpose of user responsiveness, and to assess the impact of push notifications in other health care settings.

### Conclusions

Push notifications have proven to be effective in the proposed weight loss program, leading to greater loss of fat mass and maintenance or increase of muscle mass among women who received it, specifically among those who followed a program of IPA. Future interventions should include a longer evaluation period; the impact of different message contents, as well as message delivery times and frequency, should also be researched.

## Appendix

Multimedia Appendix 1 CONSORT-EHEALTH checklist (V 1.6.1).

Multimedia Appendix 2 Main themes of the app message library.

Multimedia Appendix 3 Intranet developed for the study.

## Abbreviations

ANOVA: analysis of variance
BMI: body mass index
CONSORT: Consolidated Standards of Reporting Trials
EHEALTH: Electronic and Mobile HEalth Applications and onLine TeleHealth
IPA: intense physical activity
IPAQ-long: long version of the International Physical Activity Questionnaire
LPA: light physical activity
MPA: moderate physical activity
PA: physical activity
SMS: short message service

## References

[1] Porter J, Huggins CE, Truby H, Collins J (2016). The effect of using mobile technology-based methods that record food or nutrient intake on diabetes control and nutrition outcomes: A systematic review. Nutrients.

[2] Liu S (2019). Statista.

[3] Alonso-Arévalo J, Mirón-Canelo J (2017). Revista Cubana de Información en Ciencias de la Salud (ACIMED).

[4] (2016). Eurostat.

[5] Laxminarayan S, Istepanian RS (2000). UNWIRED E-MED: The next generation of wireless and Internet telemedicine systems. IEEE Trans Inf Technol Biomed.

[6] (2017). Business Insider.

[7] Fjeldsoe BS, Marshall AL, Miller YD (2009). Behavior change interventions delivered by mobile telephone short-message service. Am J Prev Med.

[8] Free C, Phillips G, Galli L, Watson L, Felix L, Edwards P, Patel V, Haines A (2013). The effectiveness of mobile health technology-based health behaviour change or disease management interventions for health care consumers: A systematic review. PLoS Med.

[9] Molina Recio G, García-Hernández L, Molina Luque R, Salas-Morera L (2016). The role of interdisciplinary research team in the impact of health apps in health and computer science publications: A systematic review. Biomed Eng Online.

[10] Thakkar J, Kurup R, Laba T, Santo K, Thiagalingam A, Rodgers A, Woodward M, Redfern J, Chow CK (2016). Mobile telephone text messaging for medication adherence in chronic disease: A meta-analysis. JAMA Intern Med.

[11] Redfern J, Thiagalingam A, Jan S, Whittaker R, Hackett ML, Mooney J, De Keizer L, Hillis GS, Chow CK (2014). Development of a set of mobile phone text messages designed for prevention of recurrent cardiovascular events. Eur J Prev Cardiol.

[12] Hao W, Hsu Y, Chen K, Li H, Iqbal U, Nguyen P, Huang C, Yang H, Lee P, Li M, Hlatshwayo SL, Li YJ, Jian W (2015). LabPush: A pilot study of providing remote clinics with laboratory results via short message service (SMS) in Swaziland, Africa - A qualitative study. Comput Methods Programs Biomed.

[13] Casillas J, Goyal A, Bryman J, Alquaddoomi F, Ganz P, Lidington E, Macadangdang J, Estrin D (2017). Development of a text messaging system to improve receipt of survivorship care in adolescent and young adult survivors of childhood cancer. J Cancer Surviv.

[14] Leightley D, Puddephatt J, Jones N, Mahmoodi T, Chui Z, Field M, Drummond C, Rona RJ, Fear NT, Goodwin L (2018). A smartphone app and personalized text messaging framework (InDEx) to monitor and reduce alcohol use in ex-serving personnel: Development and feasibility study. JMIR Mhealth Uhealth.

[15] Warren I, Meads A, Srirama S, Weerasinghe T, Paniagua C (2014). Push notification mechanisms for pervasive smartphone applications. IEEE Pervasive Comput.

[16] Iqbal ST, Bailey BP (2008). Effects of intelligent notification management on users and their tasks. Proceedings of the 26th Annual CHI Conference on Human Factors in Computing Systems.

[17] Pielot M, Church K, de Oliveira R (2014). An in-situ study of mobile phone notifications. Proceedings of the 16th International Conference on Human-Computer Interaction with Mobile Devices and Services.

[18] Gill S, Panda S (2015). A smartphone app reveals erratic diurnal eating patterns in humans that can be modulated for health benefits. Cell Metab.

[19] Armanasco AA, Miller YD, Fjeldsoe BS, Marshall AL (2017). Preventive health behavior change text message interventions: A meta-analysis. Am J Prev Med.

[20] Stephenson A, McDonough SM, Murphy MH, Nugent CD, Mair JL (2017). Using computer, mobile and wearable technology enhanced interventions to reduce sedentary behaviour: A systematic review and meta-analysis. Int J Behav Nutr Phys Act.

[21] Klasnja P, Pratt W (2012). Healthcare in the pocket: Mapping the space of mobile-phone health interventions. J Biomed Inform.

[22] van der Weegen S, Verwey R, Spreeuwenberg M, Tange H, van der Weijden T, de Witte L (2013). The development of a mobile monitoring and feedback tool to stimulate physical activity of people with a chronic disease in primary care: A user-centered design. JMIR Mhealth Uhealth.

[23] Falk EB, O'Donnell MB, Cascio CN, Tinney F, Kang Y, Lieberman MD, Taylor SE, An L, Resnicow K, Strecher VJ (2015). Self-affirmation alters the brain's response to health messages and subsequent behavior change. Proc Natl Acad Sci U S A.

[24] Clark JE (2015). Diet, exercise or diet with exercise: Comparing the effectiveness of treatment options for weight-loss and changes in fitness for adults (18-65 years old) who are overfat, or obese; systematic review and meta-analysis. J Diabetes Metab Disord.

[25] Golubic R, Ekelund U, Wijndaele K, Luben R, Khaw K, Wareham NJ, Brage S (2013). Rate of weight gain predicts change in physical activity levels: A longitudinal analysis of the EPIC-Norfolk cohort. Int J Obes (Lond).

[26] Liu F, Wang W, Ma J, Sa R, Zhuang G (2018). Different associations of sufficient and vigorous physical activity with BMI in Northwest China. Sci Rep.

[27] Newton RL, Carter LA, Johnson W, Zhang D, Larrivee S, Kennedy BM, Harris M, Hsia DS (2018). A church-based weight loss intervention in African American adults using text messages (LEAN Study): Cluster randomized controlled trial. J Med Internet Res.

[28] Campbell MK, DeVellis BM, Strecher VJ, Ammerman AS, DeVellis RF, Sandler RS (1994). Improving dietary behavior: The effectiveness of tailored messages in primary care settings. Am J Public Health.

[29] Bull FC, Kreuter MW, Scharff DP (1999). Effects of tailored, personalized and general health messages on physical activity. Patient Educ Couns.

[30] Walther JB, Pingree S, Hawkins RP, Buller DB (2005). Attributes of interactive online health information systems. J Med Internet Res.

[31] Bentley F, Tollmar K (2013). The power of mobile notifications to increase well-being logging behavior. Proceedings of the SIGCHI Conference on Human Factors in Computing Systems.

[32] Hagströmer M, Oja P, Sjöström M (2006). The International Physical Activity Questionnaire (IPAQ): A study of concurrent and construct validity. Public Health Nutr.

[33] Walsh JC, Corbett T, Hogan M, Duggan J, McNamara A (2016). An mHealth intervention using a smartphone app to increase walking behavior in young adults: A pilot study. JMIR Mhealth Uhealth.

[34] Thomas JG, Bond DS (2014). Review of innovations in digital health technology to promote weight control. Curr Diab Rep.

[35] Cardozo LA, Guzman C, Andrés Y, Torres M, Alejandro J (2016). Nutrición clínica y dietética hospitalaria, Volume 36, Issue 3.

[36] Forbes GB (1987). Human Body Composition: Growth, Aging, Nutrition, and Activity.

[37] Svärd A, Lahti J, Rahkonen O, Lahelma E, Lallukka T (2016). Obesity and psychotropic medication: A prospective register linkage study among midlife women and men. BMC Psychiatry.

[38] Schubert MM, Seay RF, Spain KK, Clarke HE, Taylor JK (2019). Reliability and validity of various laboratory methods of body composition assessment in young adults. Clin Physiol Funct Imaging.

[39] Callaway W, Chumlea WC, Bouchard C, Himes JH, Lohman TG, Martin AD, Mitchell CD, Mueller WH, Roche AF, Seefeldt VD, Lohman TG, Roche AF, Martorell R (1988). Circumferences. Anthropometric Standardization Reference Manual.

[40] Matthew CE (2005). Calibration of accelerometer output for adults. Med Sci Sports Exerc.

[41] Karvonen J, Vuorimaa T (1988). Heart rate and exercise intensity during sports activities: Practical application. Sports Med.

[42] Harris JA, Benedict FG (1918). A biometric study of human basal metabolism. Proc Natl Acad Sci U S A.

[43] Segal KR, Edano A, Abalos A, Albu J, Blando L, Tomas MB, Pi-Sunyer FX (1991). Effect of exercise training on insulin sensitivity and glucose metabolism in lean, obese, and diabetic men. J Appl Physiol (1985).

[44] Dietowin.

[45] Baumeister RF, Vohs KD, Tice DM (2016). The strength model of self-control. Curr Dir Psychol Sci.

[46] Polzien KM, Jakicic JM, Tate DF, Otto AD (2007). The efficacy of a technology-based system in a short-term behavioral weight loss intervention. Obesity (Silver Spring).

[47] Hebden L, Cook A, van der Ploeg HP, King L, Bauman A, Allman-Farinelli M (2014). A mobile health intervention for weight management among young adults: A pilot randomised controlled trial. J Hum Nutr Diet.

[48] Fukuoka Y, Vittinghoff E, Jong SS, Haskell W (2010). Innovation to motivation: Pilot study of a mobile phone intervention to increase physical activity among sedentary women. Prev Med.

[49] Turner-McGrievy G, Tate D (2011). Tweets, apps, and pods: Results of the 6-month Mobile Pounds Off Digitally (Mobile POD) randomized weight-loss intervention among adults. J Med Internet Res.

[50] Jacko JA (2012). Human Computer Interaction Handbook: Fundamentals, Evolving Technologies, and Emerging Applications. 3rd edition.

[51] Hutchesson MJ, Rollo ME, Krukowski R, Ells L, Harvey J, Morgan PJ, Callister R, Plotnikoff R, Collins CE (2015). eHealth interventions for the prevention and treatment of overweight and obesity in adults: A systematic review with meta-analysis. Obes Rev.

[52] Gell NM, Wadsworth DD (2015). The use of text messaging to promote physical activity in working women: A randomized controlled trial. J Phys Act Health.

[53] Fournier M, d'Arripe-Longueville F, Radel R (2017). Testing the effect of text messaging cues to promote physical activity habits: A worksite-based exploratory intervention. Scand J Med Sci Sports.

[54] Steinberg DM, Levine EL, Askew S, Foley P, Bennett GG (2013). Daily text messaging for weight control among racial and ethnic minority women: Randomized controlled pilot study. J Med Internet Res.

[55] Shapiro JR, Koro T, Doran N, Thompson S, Sallis JF, Calfas K, Patrick K (2012). Text4Diet: A randomized controlled study using text messaging for weight loss behaviors. Prev Med.

[56] Bosy-Westphal A, Schautz B, Lagerpusch M, Pourhassan M, Braun W, Goele K, Heller M, Glüer CC, Müller MJ (2013). Effect of weight loss and regain on adipose tissue distribution, composition of lean mass and resting energy expenditure in young overweight and obese adults. Int J Obes (Lond).

[57] Gallagher D, Kelley DE, Thornton J, Boxt L, Pi-Sunyer X, Lipkin E, Nyenwe E, Janumala I, Heshka S, MRI Ancillary Study Group of the Look AHEAD Research Group (2017). Changes in skeletal muscle and organ size after a weight-loss intervention in overweight and obese type 2 diabetic patients. Am J Clin Nutr.

[58] Tanaka NI, Murakami H, Aiba N, Morita A, Watanabe S, Miyachi M, Saku Control Obesity Program (SCOP) Study Group (2019). Effects of 1-year weight loss intervention on abdominal skeletal muscle mass in Japanese overweight men and women. Asia Pac J Clin Nutr.

[59] Cava E, Yeat NC, Mittendorfer B (2017). Preserving healthy muscle during weight loss. Adv Nutr.

[60] Swift DL, Johannsen NM, Lavie CJ, Earnest CP, Church TS (2014). The role of exercise and physical activity in weight loss and maintenance. Prog Cardiovasc Dis.

[61] Donnelly JE, Honas JJ, Smith BK, Mayo MS, Gibson CA, Sullivan DK, Lee J, Herrmann SD, Lambourne K, Washburn RA (2013). Aerobic exercise alone results in clinically significant weight loss for men and women: Midwest exercise trial 2. Obesity (Silver Spring).

[62] Morrison LG, Hargood C, Pejovic V, Geraghty AW, Lloyd S, Goodman N, Michaelides DT, Weston A, Musolesi M, Weal MJ, Yardley L (2017). The effect of timing and frequency of push notifications on usage of a smartphone-based stress management intervention: An exploratory trial. PLoS One.

[63] Turner-McGrievy GM, Wilcox S, Boutté A, Hutto BE, Singletary C, Muth ER, Hoover AW (2017). The Dietary Intervention to Enhance Tracking with Mobile Devices (DIET Mobile) Study: A 6-month randomized weight loss trial. Obesity (Silver Spring).

[64] Helmerhorst HJ, Brage S, Warren J, Besson H, Ekelund U (2012). A systematic review of reliability and objective criterion-related validity of physical activity questionnaires. Int J Behav Nutr Phys Act.

[65] Ferrari P, Friedenreich C, Matthews CE (2007). The role of measurement error in estimating levels of physical activity. Am J Epidemiol.

[66] Armanasco AA, Miller YD, Fjeldsoe BS, Marshall AL (2017). Preventive health behavior change text message interventions: A meta-analysis. Am J Prev Med.

