# Supplementary material for: Push Notifications From a Mobile App to Improve the Body Composition of Overweight or Obese Women: Randomized Controlled Trial
Source: JMIR Mhealth Uhealth. 2020 Feb 12;8(2):e13747. doi: 10.2196/13747 (PMC7055755; doi:10.2196/13747)
Supplement: Multimedia Appendix 2 [file mhealth_v8i2e13747_app2.pptx]

## Slide 1
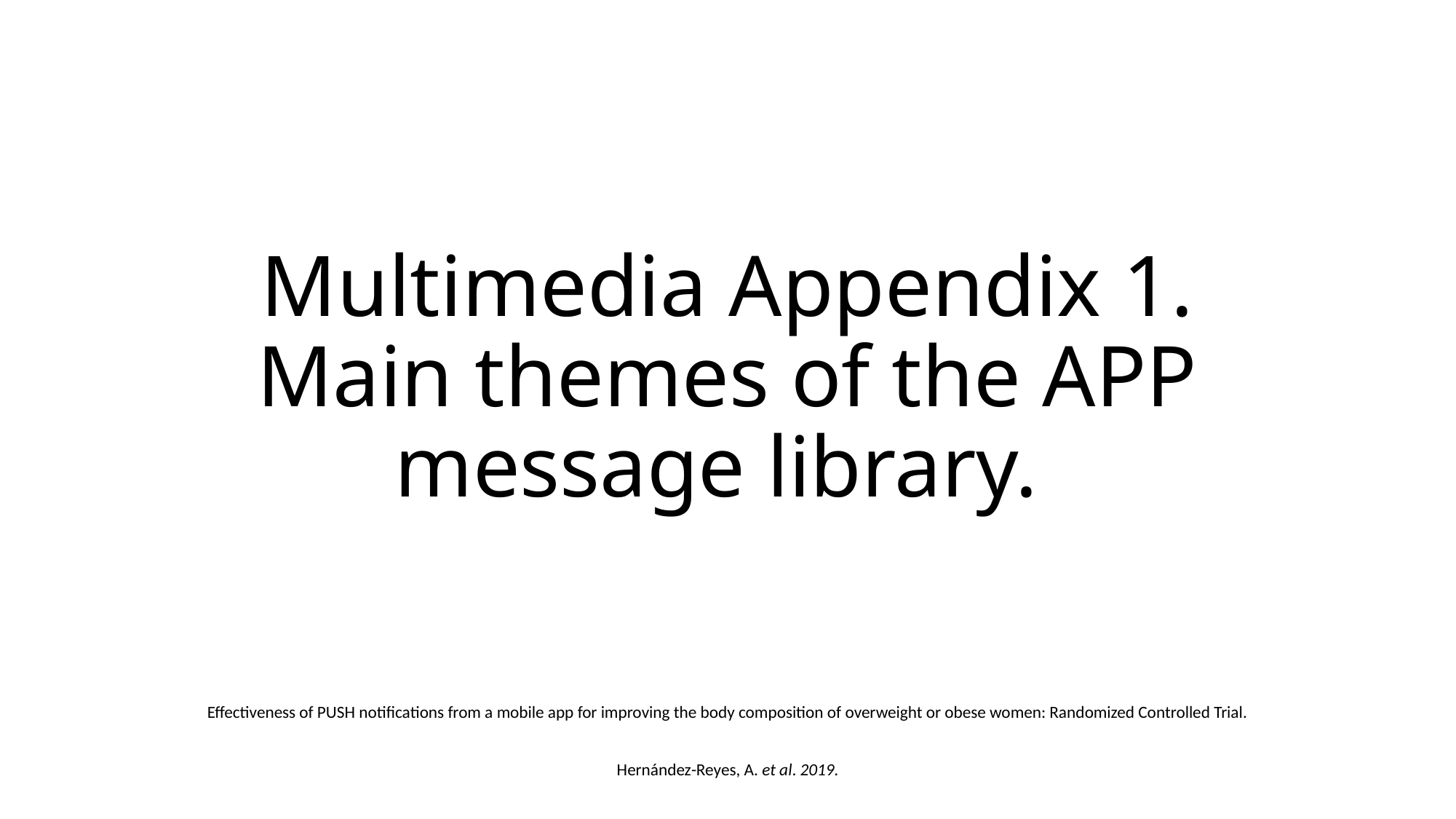

# Multimedia Appendix 1. Main themes of the APP message library.
Effectiveness of PUSH notifications from a mobile app for improving the body composition of overweight or obese women: Randomized Controlled Trial.
Hernández-Reyes, A. et al. 2019.

## Slide 2
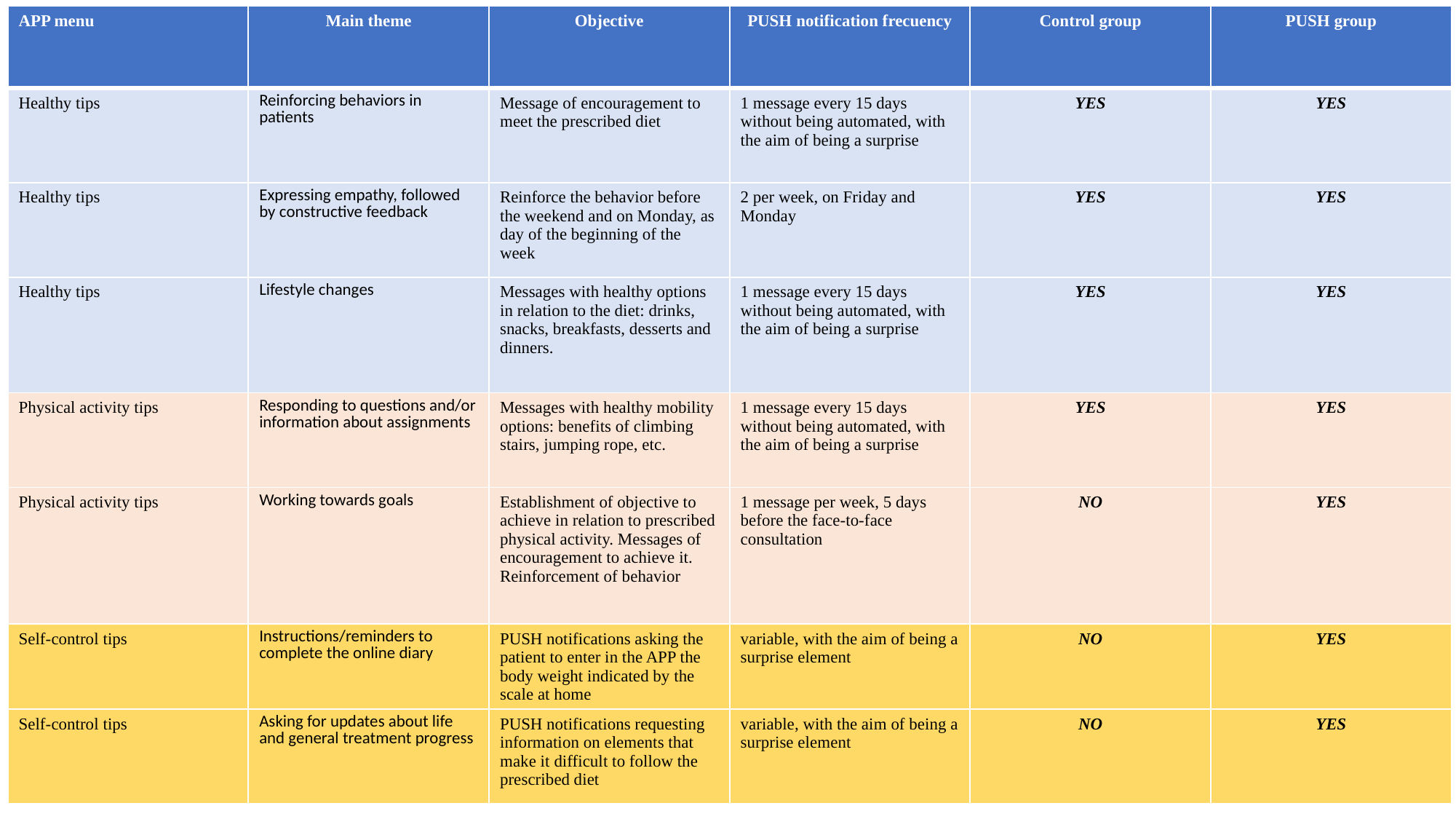

| APP menu | Main theme | Objective | PUSH notification frecuency | Control group | PUSH group |
| --- | --- | --- | --- | --- | --- |
| Healthy tips | Reinforcing behaviors in patients | Message of encouragement to meet the prescribed diet | 1 message every 15 days without being automated, with the aim of being a surprise | YES | YES |
| Healthy tips | Expressing empathy, followed by constructive feedback | Reinforce the behavior before the weekend and on Monday, as day of the beginning of the week | 2 per week, on Friday and Monday | YES | YES |
| Healthy tips | Lifestyle changes | Messages with healthy options in relation to the diet: drinks, snacks, breakfasts, desserts and dinners. | 1 message every 15 days without being automated, with the aim of being a surprise | YES | YES |
| Physical activity tips | Responding to questions and/or information about assignments | Messages with healthy mobility options: benefits of climbing stairs, jumping rope, etc. | 1 message every 15 days without being automated, with the aim of being a surprise | YES | YES |
| Physical activity tips | Working towards goals | Establishment of objective to achieve in relation to prescribed physical activity. Messages of encouragement to achieve it. Reinforcement of behavior | 1 message per week, 5 days before the face-to-face consultation | NO | YES |
| Self-control tips | Instructions/reminders to complete the online diary | PUSH notifications asking the patient to enter in the APP the body weight indicated by the scale at home | variable, with the aim of being a surprise element | NO | YES |
| Self-control tips | Asking for updates about life and general treatment progress | PUSH notifications requesting information on elements that make it difficult to follow the prescribed diet | variable, with the aim of being a surprise element | NO | YES |

## Slide 3
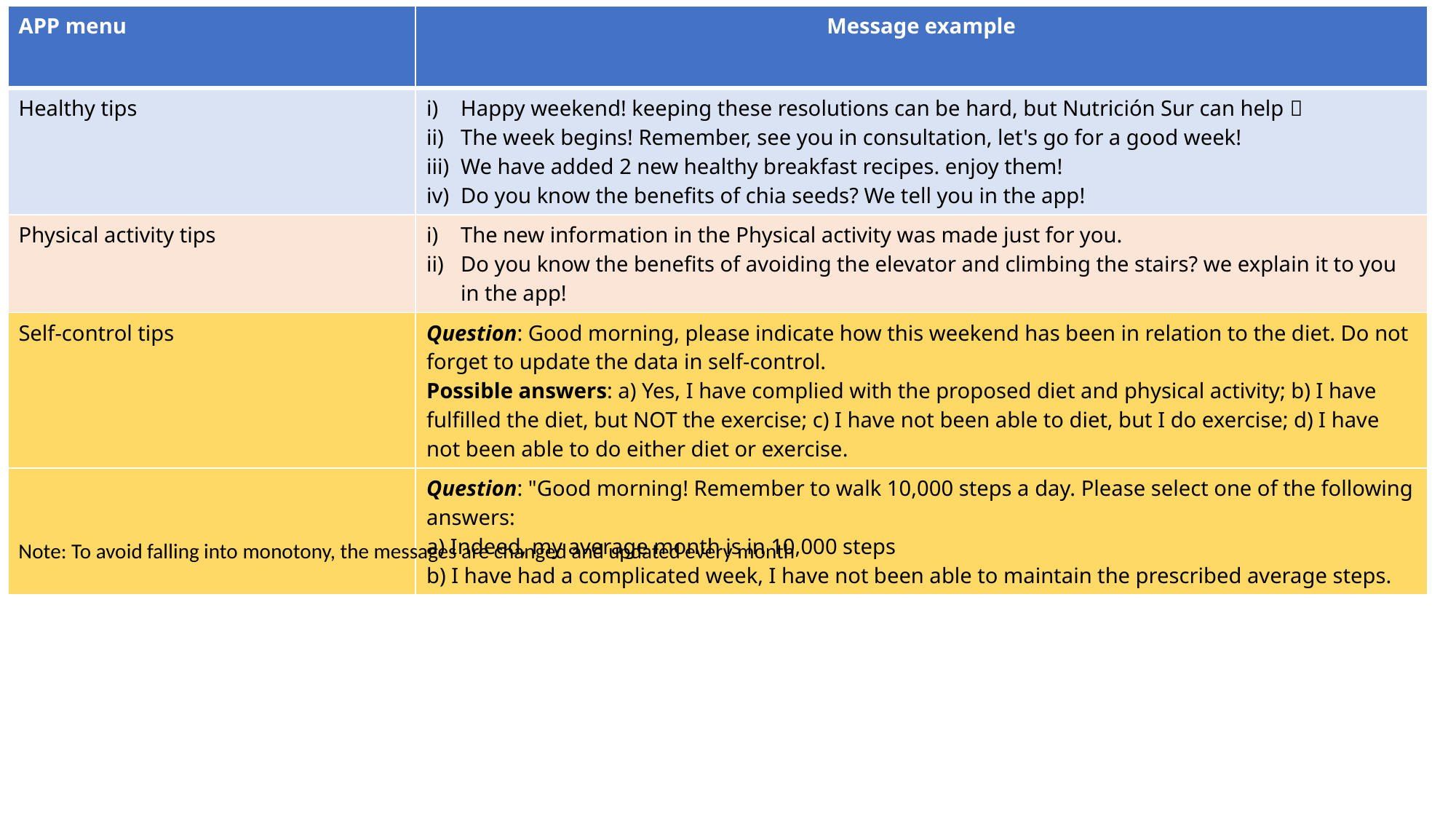

| APP menu | Message example |
| --- | --- |
| Healthy tips | Happy weekend! keeping these resolutions can be hard, but Nutrición Sur can help  The week begins! Remember, see you in consultation, let's go for a good week! We have added 2 new healthy breakfast recipes. enjoy them! Do you know the benefits of chia seeds? We tell you in the app! |
| Physical activity tips | The new information in the Physical activity was made just for you. Do you know the benefits of avoiding the elevator and climbing the stairs? we explain it to you in the app! |
| Self-control tips | Question: Good morning, please indicate how this weekend has been in relation to the diet. Do not forget to update the data in self-control. Possible answers: a) Yes, I have complied with the proposed diet and physical activity; b) I have fulfilled the diet, but NOT the exercise; c) I have not been able to diet, but I do exercise; d) I have not been able to do either diet or exercise. |
| | Question: "Good morning! Remember to walk 10,000 steps a day. Please select one of the following answers: a) Indeed, my average month is in 10,000 steps b) I have had a complicated week, I have not been able to maintain the prescribed average steps. |
Note: To avoid falling into monotony, the messages are changed and updated every month
