# Supplementary material for: Push Notifications From a Mobile App to Improve the Body Composition of Overweight or Obese Women: Randomized Controlled Trial
Source: JMIR Mhealth Uhealth. 2020 Feb 12;8(2):e13747. doi: 10.2196/13747 (PMC7055755; doi:10.2196/13747)
Supplement: Multimedia Appendix 3 [file mhealth_v8i2e13747_app3.pdf]

Multimedia Appendix 2. Intranet developed for the study

Effectiveness of PUSH notifications from a mobile app for improving the body composition of overweight or obese women: Randomized Controlled Trial.

Hernández-Reyes, A. *et al*. *2019*.

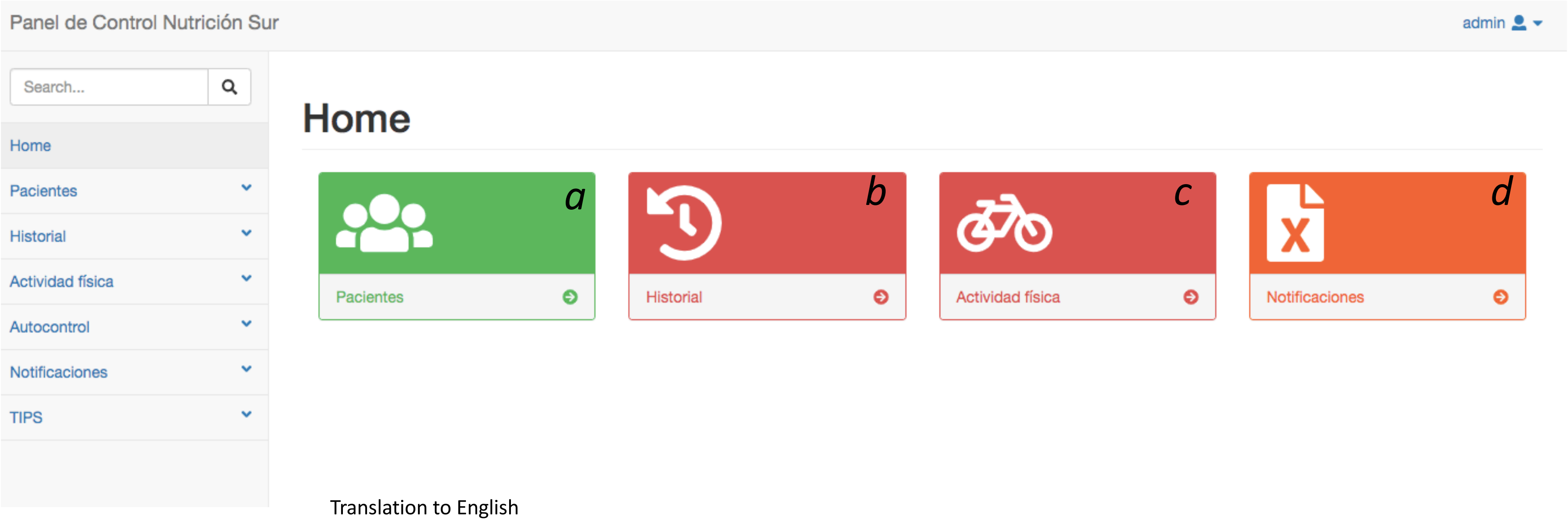

Translation to English

a) Patients
b) Clinical history
c) Physical activity
d) PUSH notifications

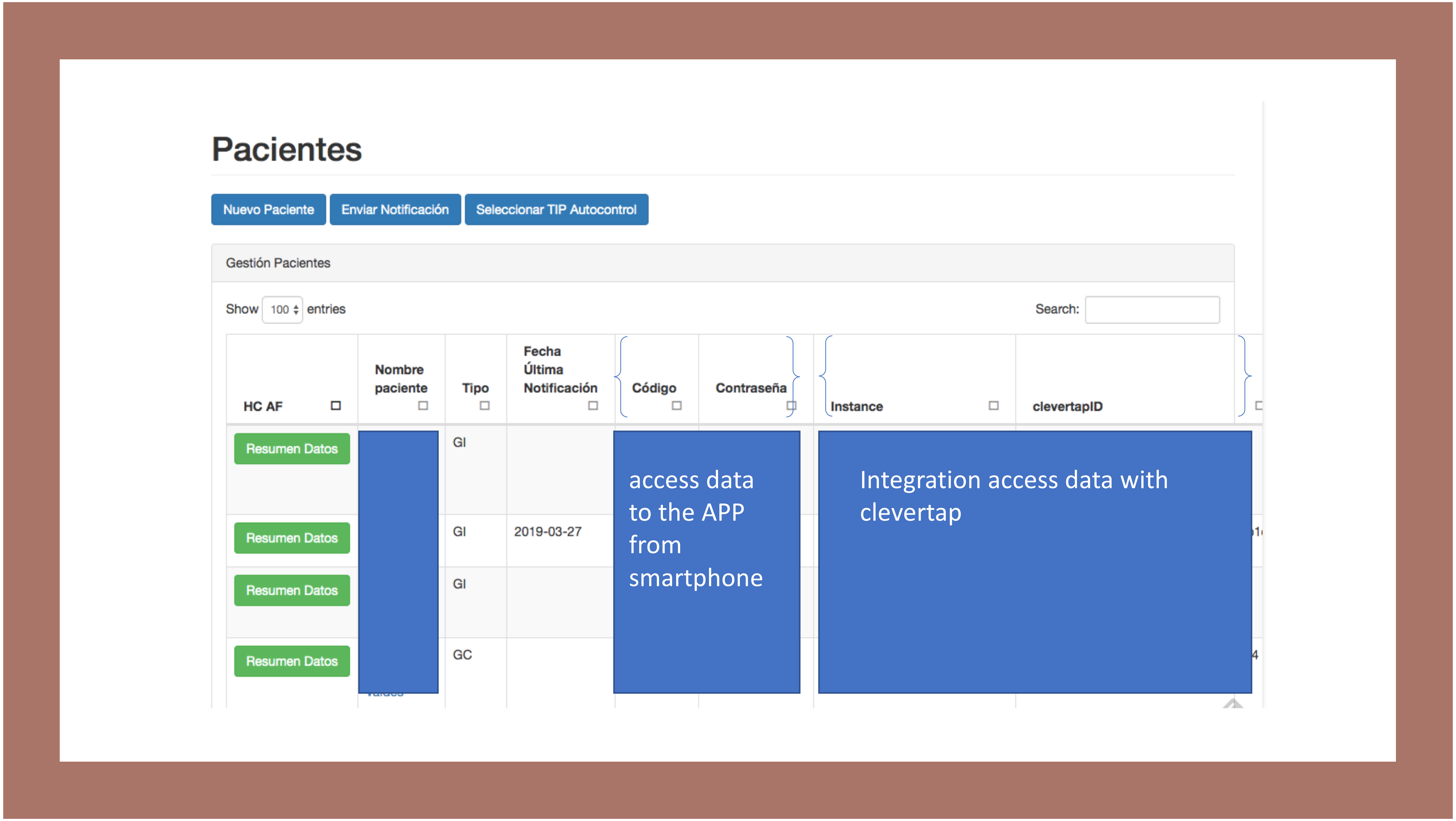
Pacientes
Nuevo Paciente
Enviar Notificación
Seleccionar TIP Autocontrol
Gestión Pacientes
Show 100 entries
Search:
HC AF
Nombre paciente
Tipo
Fecha Última Notificación
Código
Contraseña
Instance
clevertapID
Resumen Datos
GI
Resumen Datos
GI
2019-03-27
Resumen Datos
GI
Resumen Datos
GC
access data to the APP from smartphone
Integration access data with clevertap

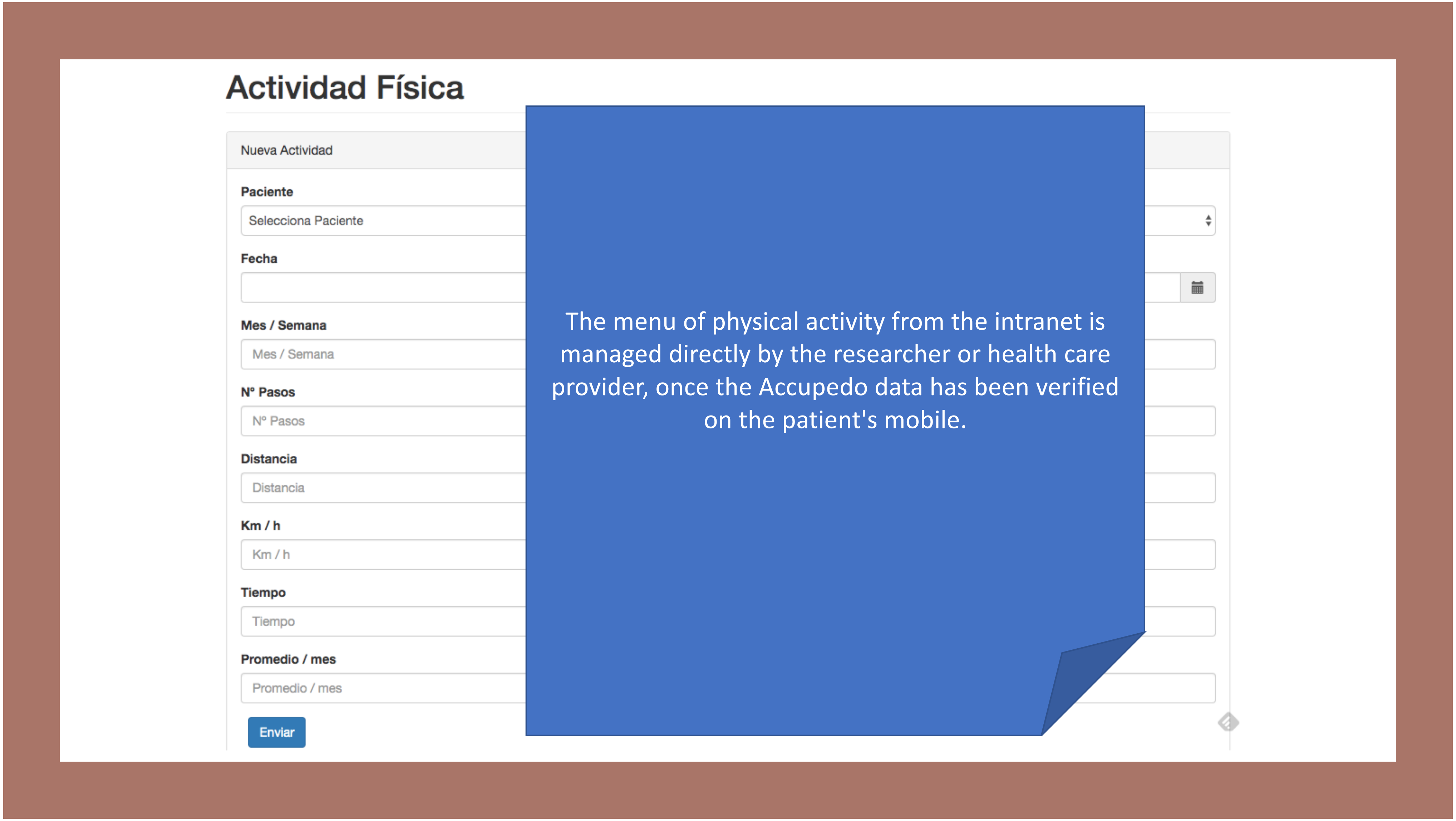
Actividad Física
Nueva Actividad
Paciente
Selecciona Paciente
Fecha
Mes / Semana
Mes / Semana
Nº Pasos
Nº Pasos
Distancia
Distancia
Km / h
Km / h
Tiempo
Tiempo
Promedio / mes
Promedio / mes
Enviar
The menu of physical activity from the intranet is managed directly by the researcher or health care provider, once the Accupedo data has been verified on the patient's mobile.

Autocontrol

The AUTOCONTROL menu allows the researcher and health professional to access the following information: prescribed diet fulfilled yes or no, physical activity prescribed yes or no and body weight at home (measured on the home scale). This information is used in the intervention group.

Datos enviados

Show 100 entries

Search: [redacted]

fecha	Paciente	Dieta	Actividad Física	Peso	
2019-03-21	[redacted]	no	si	64,2	
2019-03-06	[redacted]	no	no	64,6	
2019-03-06	[redacted]	no	no	64,6	
2019-02-27	[redacted]	si	si	64,7	
2019-02-27	[redacted]	si	si	64,7	
2019-02-27	[redacted]	si	si	64,7	
2019-02-20	[redacted]	si	si	65	
2019-02-20	[redacted]	si	si	65	
2019-02-13	[redacted]	si	si	64,7	
2019-02-13	[redacted]	si	si	64,7	

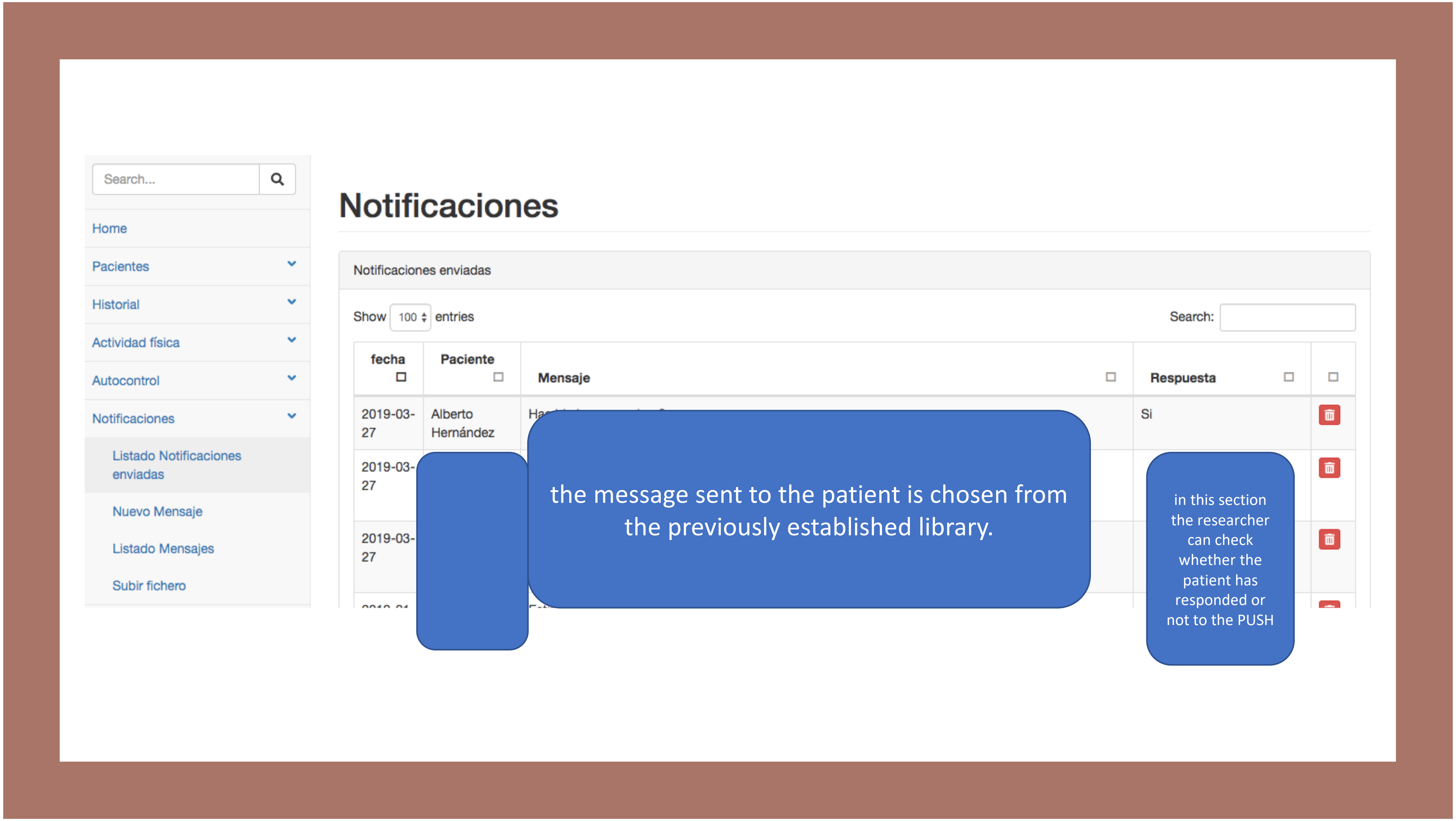
Search...
Home
Pacientes
Historial
Actividad física
Autocontrol
Notificaciones
Listado Notificaciones enviadas
Nuevo Mensaje
Listado Mensajes
Subir fichero
Notificaciones
Notificaciones enviadas
Show 100 entries
Search:
fecha
Paciente
Mensaje
Respuesta
2019-03-27
Alberto Hernández
Si
2019-03-27
2019-03-27
the message sent to the patient is chosen from the previously established library.
in this section the researcher can check whether the patient has responded or not to the PUSH

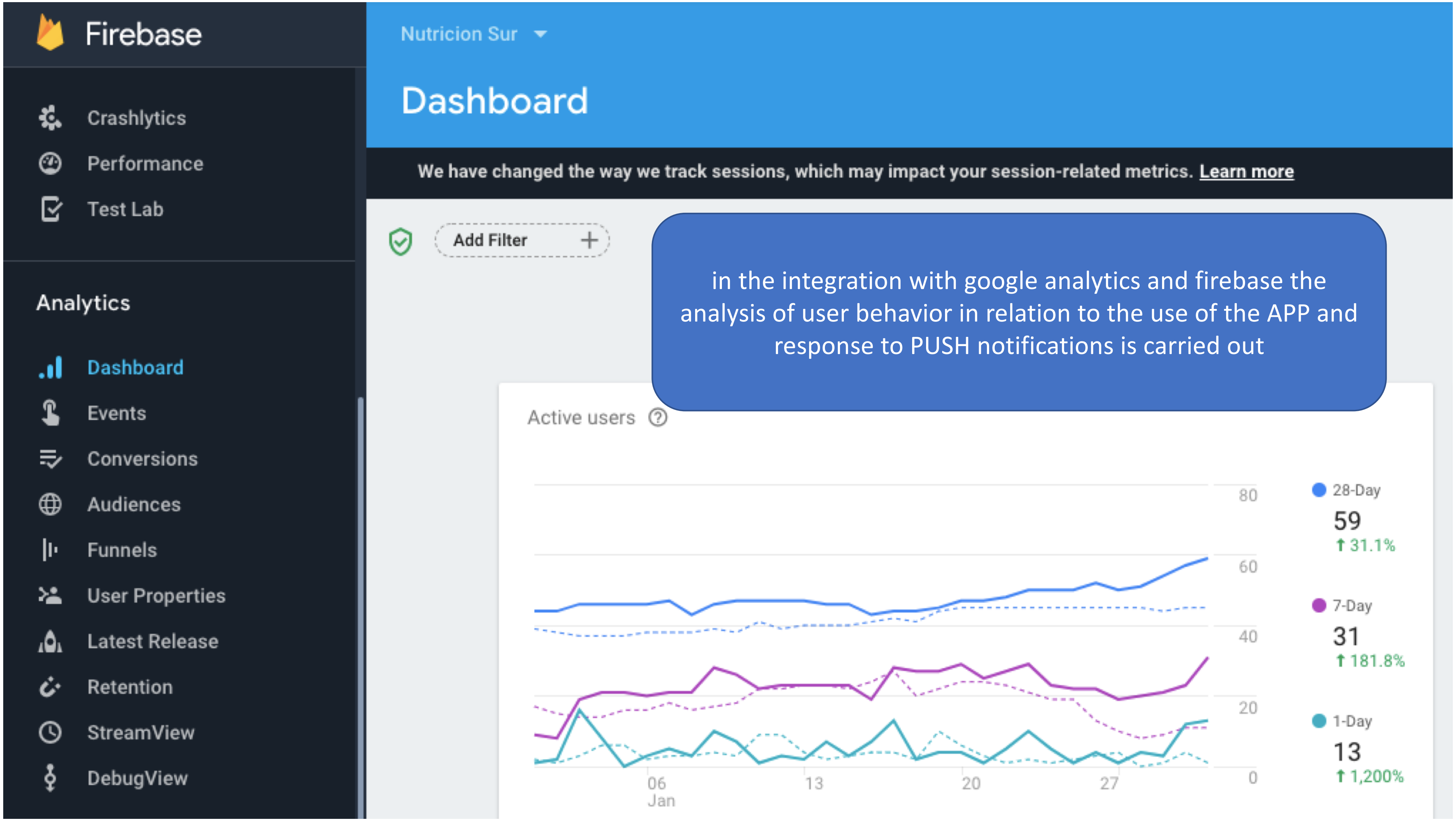

Firebase
Crashlytics
Performance
Test Lab
Analytics
Dashboard
Events
Conversions
Audiences
Funnels
User Properties
Latest Release
Retention
StreamView
DebugView
Nutricion Sur
Dashboard
We have changed the way we track sessions, which may impact your session-related metrics. Learn more
Add Filter
in the integration with google analytics and firebase the analysis of user behavior in relation to the use of the APP and response to PUSH notifications is carried out
Active users
80
60
40
20
0
06 Jan
13
20
27
28-Day
59
31.1%
7-Day
31
181.8%
1-Day
13
1,200%
